# Construction and Characterization of MoClo-Compatible Vectors for Modular Protein Expression in *E. coli*

**DOI:** 10.1021/acssynbio.4c00564

**Published:** 2025-01-13

**Authors:** Jochem R. Nielsen, Michael J. Lewis, Wei E. Huang

**Affiliations:** Department of Engineering Science, University of Oxford, Oxford OX1 3PJ, U.K.

**Keywords:** golden gate assembly, MoClo, origin of replication, destination vectors, GFP expression, plasmid burden, growth-coupled selection, plasmid, copy number

## Abstract

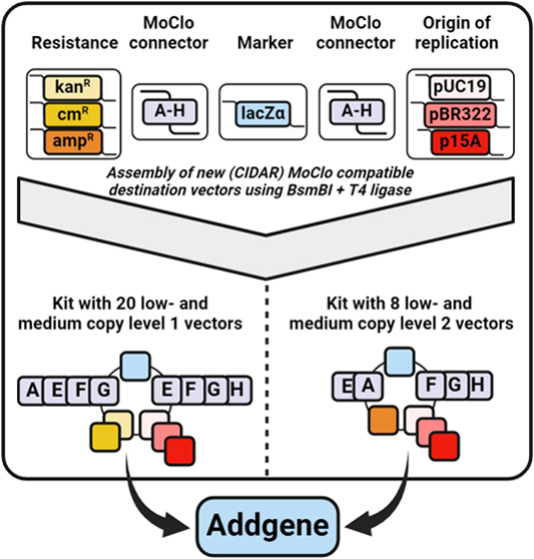

Cloning methods are fundamental to synthetic biology research. The capability to generate custom DNA constructs exhibiting predictable protein expression levels is crucial to the engineering of biology. Golden Gate cloning, a modular cloning (MoClo) technique, enables rapid and reliable one-pot assembly of genetic parts. In this study, we expand on the existing MoClo toolkits by constructing and characterizing compatible low- (p15A) and medium-copy (pBR322) destination vectors. Together with existing high-copy vectors, these backbones enable a protein expression range covering a 500-fold difference in normalized fluorescence output. We further characterize the expression- and burden profiles of each vector and demonstrate their use for the optimization of growth-coupled enzyme expression. The optimal expression of *adhE* (encoding alcohol dehydrogenase) for ethanol-dependent growth of *Escherichia coli* is determined using randomized Golden Gate Assembly, creating a diverse library of constructs with varying expression strengths and plasmid copy numbers. Through selective growth experiments, we show that relatively low expression levels of *adhE* facilitated optimal growth using ethanol as the sole carbon source, demonstrating the importance of adding low-copy vectors to the MoClo vector repertoire. This study emphasizes the importance of varying vector copy numbers in selection experiments to balance expression levels and burden, ensuring accurate identification of optimal conditions for growth. The vectors developed in this work are publicly available via Addgene (catalog #217582–217609).

## Introduction

The field of synthetic biology is built upon the ability to manipulate DNA sequences rapidly and reliably. The availability of genetic toolkits to predictably manipulate protein expression levels has allowed biology to become increasingly engineered. Establishing genetic circuitry relies on the correct assembly of functional genetic regions including promoters, ribosome binding sites (RBS), coding DNA sequences (CDS), and terminators, allowing the construction of custom DNA sequences with designed functionalities.

Effective DNA assembly methods are essential tools for any synthetic biologist. These assembly techniques include traditional restriction enzyme cloning, such as blunt-end or sticky-end cloning, and seamless cloning methods such as Gibson assembly.^1^ Although cloning using restriction enzymes has been common practice for decades, this technique was modernized with the conception of BioBricks.^2^ This method relied on the use of a set of standardized restriction sites to enable the modular assembly of characterized DNA fragments. The standardized assembly methods, modular parts repositories, and most importantly its widespread adoption by the synthetic biology community represented a leap forward in the field. However, BioBricks suffered from the same technical limitations as traditional restriction enzyme cloning, most notably low efficiencies with complex assemblies.

In 2008, a new DNA assembly method coined Golden Gate Assembly (GGA) was introduced, which was based on Type IIS restriction enzymes displaying restriction activity distal from their own recognition site.^3^ This activity removes the enzyme’s own recognition site after restriction, a principle that can be exploited to drastically improve assembly efficiencies by cycling between restriction- and ligation steps to form increasing correctly assembled product within a reaction mix after each cycle.^3−5^ Indeed, correct assemblies with up to 52 parts covering 40 kb have been reported using GGA.^6^ In addition, GGA allows the use of both circular- and linear DNA as parts simultaneously, expanding the method’s flexibility. The most commonly used Type IIS enzymes (BsaI, BbsI, BsmBI/Bpil) leave 4 bp sticky overhangs, but a GGA kit using SapI leaving 3 bp overhangs has also been developed.^7^

Since the inception of GGA, many genetic part toolkits have been characterized and made publicly available for use in plants,^8,9^ yeast,^10^ fungi,^11^ cyanobacteria^12^ and those characterized more specifically for *Vibrio natriegens*,^13^*Pseudomonas putida*,^14^ and *Escherichiacoli*.^7,15−17^ Efforts to establish unified cross-genus or cross-kingdom GGA cloning standards are also underway.^18−20^ For *E. coli*, the CIDAR MoClo kit was the first modular Golden Gate DNA parts library made publicly available via Addgene (Kit #1000000059) and it has been widely used since.^17^ This kit contained a series of promoters and RBSs from the Anderson collection, as well as several inducible promoter parts, their repressors, and CDS parts coding for fluorescent proteins. All parts and destination vectors in the CIDAR MoClo kit were constructed using vectors with high-copy pUC19-derived origins of replication, but the kit lacked medium- or low copy destination vectors. For the purpose of expressing whole metabolic pathways or large genetic constructs, high copy vectors can become burdensome or lethal to *E. coli*, limiting the use of the kit. A variety of different plasmid backbones can be hosted by *E. coli*, exhibiting variable plasmid copy numbers based on their mode of replication or the presence of mutations.^21,22^

In this study, we describe the construction and characterization of a MoClo-compatible vector kit for use in *E. coli*. We generated vectors for level 1 assembly carrying either p15A- (low copy) or pBR322-derived (medium copy) origins of replication with either kanamycin or chloramphenicol resistance. Level 2 destination vectors were designed with ampicillin resistance and also carry either p15A- or pBR322-derived origins of replication. Since the p15A origin of replication is compatible with the pMB1-based pBR322- or pUC19 origins, this vector set enables the modular construction of dual plasmid systems in *E. coli*. Combined with the existing pUC19 vectors, the plasmids in this kit enable an exploration of the protein expression space covering a 500-fold difference in normalized GFP expression. In addition, we demonstrated the use of these plasmids for optimizing enzyme expression using growth-coupled selection. We expect that this kit could be of use to synthetic biologists using Golden Gate cloning in *E. coli*, especially for the expression of metabolic pathways, burdensome constructs, or dual-plasmid systems. The plasmids constructed in this work are publicly available via Addgene (catalog #217582–217609).

## Materials and Methods

### Strains, Plasmids, Media, and Growth Conditions

The plasmids used and constructed in this work are listed in Table S1. The primers used in this study are provided in Table S2, while the synthesized DNA for vector construction, detailed later, is available in Table S3. The strains used for this study Table 1. Routine cloning was carried out using*E. coli* DH5α (NEB). Transformations were typically performed via heat shock, with growth carried out in LB medium at 250 rpm and 37 °C. Cells were supplemented with appropriate antibiotics where necessary: carbenicillin (carb, 100 μg/mL); chloramphenicol (cm, 25 μg/mL); kanamycin (kan, 50 μg/mL). For blue-white screening, LB agar plates were supplemented with 100 μg/mL 5-bromo-4-chloro-3-indolyl-β-d-galactopyranoside (X-gal, Sigma) and 100 μM isopropyl β-d-1-thiogalactopyranoside (IPTG, Sigma). Modified minimal M9 medium was used as indicated in the text, comprised of 47.8 mM Na_2_HPO_4_, 22 mM KH_2_PO_4_, 8.6 mM NaCl, 18.7 mM NH_4_Cl, 2 mM MgSO_4_, 100 μM CaCl_2_, 134 μM ethylene diamine tetrachloroacetic acid (EDTA), 31 μM FeCl_3_·6H_2_O, 6.2 μM ZnCl_2_, 0.76 μM CuCl_2_·2H_2_O, 0.42 μM CoCl_2_·2H_2_O, 1.62 μM H_3_BO_3_, and 0.081 μM MnCl_2_·4H_2_O, adjusted to pH 7.0. A final concentration of 1% w/v filter sterilized d-glucose was added as a sole carbon source (Table 1).

**Table 1:** Strains Used and Constructed in This Work

strain	genotype	reference
E. coli DH5α	*fhuA2Δ(argF-lacZ)U169 phoA gln V44 Φ80*Δ*(lacZ)M15 gyrA96 recA1 relA1 endA1 thi-1 hsdR17*	purchased (New England Biolabs)
BW25113	*F-LAM- rrnB3 ΔlacZ4787 hsdR514 Δ(araBAD)567* Δ*(rhaBAD)568 rph-1*	purchased (DSMZ)
BW25113 Δ*adhE*	BW25113 Δ*adhE::FRT*	this study

### Assembly of the MoClo Destination Vectors

Resistance markers (chloramphenicol, kanamycin, and ampicillin), origins of replication (p15A and pBR322) and the expression construct for lacZα were synthesized as double strand DNA fragments (Twist, USA) with BsmBI-digestible flanks (Table S3). Kanamycin- and ampicillin markers were synthesized identically to those found in DVA and DVK vectors, while the chloramphenicol marker was synthesized based on the sequence found in pKD3 but modified to carry a silent G435T mutation for domestication. Short DNA oligos introducing MoClo compatible overhangs A–H and containing the required BsaI and BbsI recognition sites were designed as two complementary oligos with BsmBI-digestible flanks (Table S2, JRN413–440). The oligo design differed depending on their use for level 1 or level 0/2 constructs, since BsaI and BbsI recognition sites had to be inverted accordingly. The synthesized fragments and oligos were designed to be assembled using GGA, with complementary overhangs between resistance marker and ori fragments (GATT, “X”), resistance marker fragment and oligo (TGGA, “T”), ori fragment and oligo (TTCT, “U”), oligo and one flank of the lacZα fragment (CCTG, “V”), and oligo with the other flank of the lacZα fragment (GGGT, “S”). Single stranded complementary oligos were annealed by combining 100 μM of each primer in 50 μL deionized H_2_O and heating to 98 °C for 5 min, followed by cooling to 25 °C at 0.1 °C/s using a thermocycler. Destination vectors were generated by combining 20 fmol of the antibiotic resistance marker, origin of replication and lacZα expression fragments, in addition to 1 μL of each 10×-diluted annealed oligo containing the desired MoClo overhangs. Golden Gate assemblies were performed in a total of 20 μL with 2 μL of 10× T4 DNA ligase buffer (NEB), 10 units of BsmBI-HF (NEB) and 500 units of T4 DNA ligase (NEB). Reaction mixtures were cycled between incubations for 5 min at 42 °C and 5 min at 16 °C for a total of 80 cycles in a thermocycler, with two final 10 min steps at 42 and 80 °C before cooling to 4 °C. A 4 μL aliquot of the assembly mixture was transformed into*E. coli* DH5α and correct assemblies were identified by the formation of blue*E. coli* colonies on LB supplemented with the required antibiotic, X-gal and IPTG. Plasmids were extracted (Monarch Plasmid Miniprep Kit, NEB) and constructs were verified by Sanger sequencing (Eurofins, UK) using primers JRN273 and JRN274.

### Routine Golden Gate Assemblies

Routine Golden Gate cloning was performed mostly as described earlier.^17^ Briefly, for level 1 assemblies including the GFP constructs used in this study, 20 fmol of destination vector was mixed with 40 fmol of each relevant part consisting of a promoter, RBS, CDS, and terminator. All parts can be found in Table S1. Enzyme/buffer concentrations and cycling conditions were identical to those used for the destination vector assemblies, except BsmBI-HF was replaced by BsaI-HFv2 (NEB) and a restriction temperature of 37 °C was used instead of 42 °C.

### Gene Deletion of *adhE* in*E. coli* BW25113

The deletion of *adhE* was performed by lambda red recombination mostly according to previous work.^23^ Briefly, *E. coli* BW25113 was transformed with pSIJ8, encoding the lambda red recombination and flippase genes. Transformants were grown selectively overnight at 30 °C and 1 mL of O/N culture was used to inoculate 50 mL of LB + carb in a shake flask. This culture was left to grow for 1 h at 30 °C, followed by pSIJ8 induction for 45 min by addition of sterile 15 mM l-arabinose. Cells were made electrocompetent by washing twice in 35 mL ice-cold dH_2_O and finally resuspended in 300 μL ice-cold sterile 10% glycerol. DNA cassettes targeting *adhE* were generated by PCR amplification of pKD3 with JRN384 and JRN385 (Table S2). DNA was purified (PCR Cleanup Kit, NEB) and eluted in DI water. For electroporation of the marker, 50 μL of electrocompetent cells were mixed with 3.5 μL of marker (>250 ng/μL) and transferred to an ice-cold electroporation cuvette (0.1 cm gap). A 1.8 kV pulse was applied, and cells were recovered immediately for 2.5 h at 30 °C before plating on LB + carb + cm (17 μg/mL) and incubated at 30 °C. Positive clones were identified by colony PCR of the *adhE* locus using primers JRN386 and JRN387. Integrated chloramphenicol markers were removed by inoculating 35 mL of LB + carb with 0.5 mL of overnight*E. coli* BW25113 *adhE::cmR* pSIJ8 culture. After 1.5 h shaking at 30 °C, flippase expression was induced with 50 mM L-rhamnose for 4 h before plating 10^–5^ and 10^–6^ dilutions on LB + carb plates. Strains with *adhE::FRT* genotypes (Δ*adhE*) were identified using the same *adhE* colony PCR primers and pSIJ8 was cured by a single overnight cultivation in LB at 42 °C.

### Domestication of *adhE* and Randomized adhE* Construct Assembly

The*E. coliadhE* gene carries several endogenous BbsI recognition sites which had to be removed prior to its use as a Golden Gate CDS part. Genomic DNA of*E. coli* MG1655 (carries an identical *adhE* nucleotide sequence to*E. coli* BW25113) was used as a template for amplification with primers JRN279 + JRN280, JRN281 + JRN282, JRN283 + JRN284, JRN285 + 286 and JRN287 + 288 to generate five linear fragments of the *adhE* gene with silent mutations removing the internal BbsI recognition sites. All fragments were designed with overhangs including BbsI recognition sites to enable one-step assembly of the domesticated parts into DVA_pUC19_CD, resulting in pL0_JRN012_CD. The A267T mutation was introduced by amplifying the domesticated level 0 *adhE* part from pL0_JRN012_CD using primers JRN224 + JRN225 and performing a Gibson assembly (NEBuilder HiFi DNA Assembly Master Mix, NEB) using an oligo “bridge” (JRN226) between the two flanks, introducing the mutation and forming pL0_JRN017_CD. The E568K mutation was introduced in the same way but using pL0_JRN017_CD as PCR template and using primers JRN189 + JRN190 with oligo bridge JRN191, forming pL0_JRN018_CD now containing *adhE*^A267T/E568 K^ (adhE*). For generation of an adhE* expression library, 6.67 fmol of DVK_pUC19_AE, DVK_pBR322_AE and DVK_p15A_AE were added to the Golden Gate reaction mix, along with 40 fmol of promoter part, RBS part, pL0_JRN018_CD, and terminator part (see text for promoter/RBS specification). Enzyme/buffer concentrations and cycling conditions were identical to those described earlier in the Methods. A 4 μL aliquot of the assembly mix was transformed into*E. coli* DH5α, recovered for 1h and an aliquot plated on LB + IPTG + X-gal + kan to assess library fidelity. The remaining recovered culture was washed twice in fresh LB to remove nontransformed DNA, resuspended in 5 mL of LB + kan and grown overnight for plasmid isolation the following day (NEB).

### Selection of adhE* Expression Constructs

Three separate 50 μL aliquots of electrocompetent BW25113 Δ*adhE* were transformed by electroporation as described earlier with 1 μL of plasmid DNA library carrying randomized adhE* expression constructs. Each transformed culture was recovered immediately by incubating in LB at 37 °C for 1 h. After recovery, 50 μL of each replicate was plated on LB + kan to assess the diversity of the library pre-selection. The remaining recovered cells were washed three times in sterile 1× M9 salts and each replicate was used to inoculate a separate 250 mL flask containing 50 mL of M9 minimal medium supplemented with 1 mM glucose, 300 mM ethanol and 50 μg/mL kanamycin. The flasks were placed in a 37 °C shaking incubator (225 rpm) and OD_600_ readings were taken daily. When OD_600_ values reached > 1.0, 1 mL of grown culture was diluted 10^4^- and 10^5^-fold and 100 μL was plated on LB + kan plates. The grown culture was then passaged by diluting it 100-fold into fresh 50 mL of M9 medium supplemented as before, for two additional passages. Plasmid contents of clones were assessed by Sanger sequencing (Source Bioscience, UK) of amplified vector inserts (DreamTaq, ThermoFisher) using primers JRN273 and JRN274. Four colonies from each replicate population were sequenced at every step (pre-selection and passages 1, 2, and 3).

### Microtiter Plate Assays

Microbial growth and fluorescence were assessed in a Tecan Spark plate reader (SparkControl v3.1 SP1 software), equipped with a humidity cassette. Assays were performed in 200 μL LB or M9 medium as indicated in the text and wells were sealed with Breathe Easy (Sigma-Aldrich) membranes to avoid contamination. Growth took place at 37 °C and measured by absorbance at 600 nm (OD_600_) while GFP fluorescence was assessed by monochromatic excitation at 480 nm and emission at 520 nm, using 30 flashes, a 20 nm bandwidth for both emission- and excitation, a 40 μs integration time, and a Z-position of 30 mm (measurement were taken from the top of the plate). A manual gain setting of 60 (A.U.) was used for fluorescence readings. Readings were taken every 15 min with agitation in between readings occurring in four separate stages: (1) 225 s, 3 mm amplitude, 90 rpm; (2) 225 s, 3 mm amplitude, 180 rpm; (3) 225 s, 2.5 mm amplitude, 108 rpm; (4) 225 s, 2.5 mm amplitude, 216 rpm. Normalized fluorescence values were calculated by dividing the measured relative fluorescence units (RFU) by the OD_600_ of the same population. Growth rates were calculated through a log conversion of the blank-medium corrected OD_600_ values, followed by determining the maximum slope of a linear regression over these values using a 6–12 h sliding window per replicate.

## Results

### Expression and Burden Assessment of the Vector Kit

**Figure 1:**
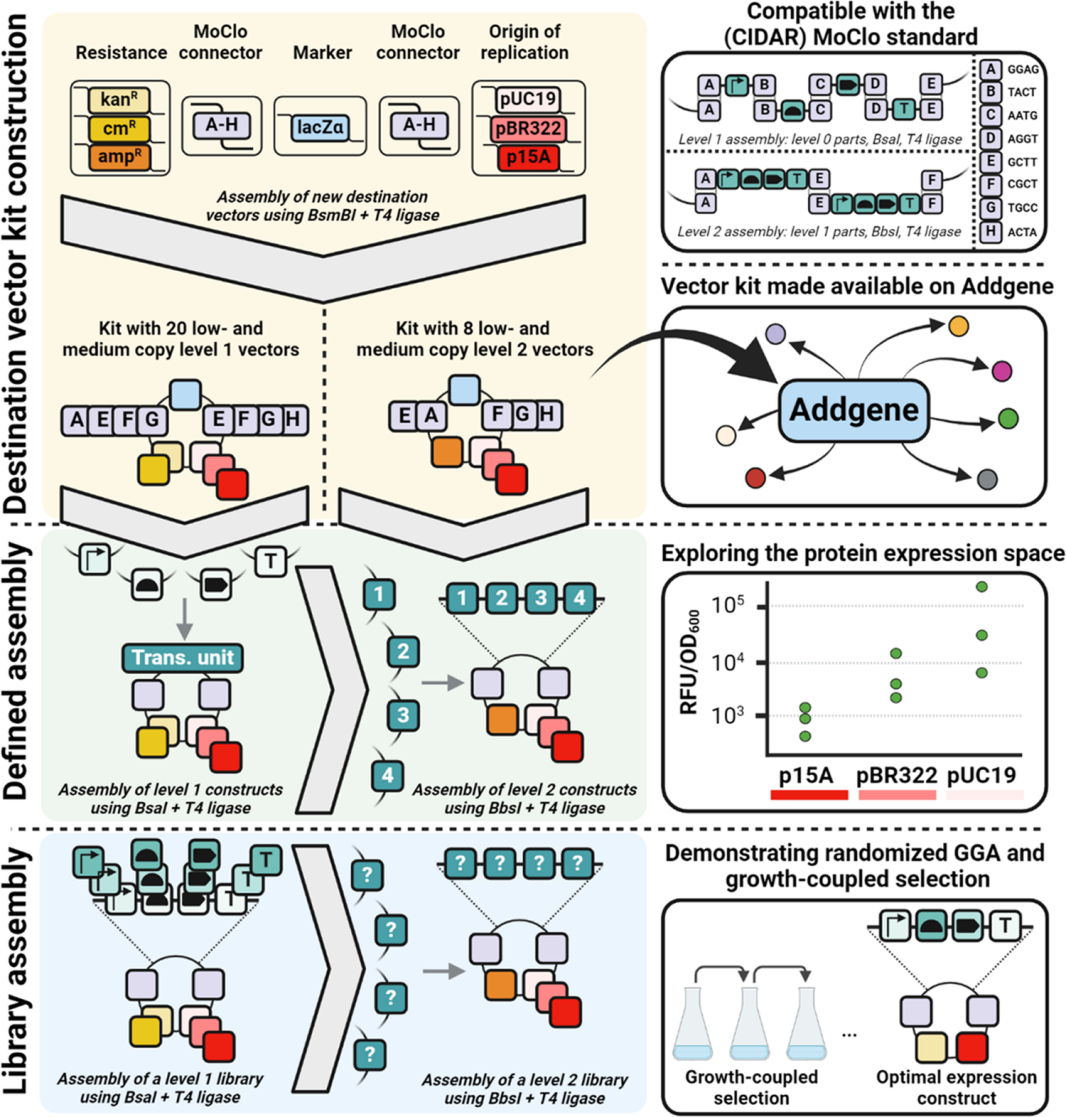
Schematic overview of modular cloning and the contributions of this study. To facilitate Golden Gate Assembly with low- and medium copy plasmids, a kit containing 20 level one and 8 level two destination vectors was constructed (yellow background), carrying either p15A or pBR322-derived origins of replication. These destination vectors can be used for defined assembly of expression constructs (green background). The effect of the origin of replication, promoter, and RBS on GFP expression was characterized in this work to allow effective deployment of this kit, which has been made available on Addgene. The addition of these new low- and medium copy number destination vectors can be used to assemble libraries of expression constructs (blue background). Using growth-coupled selection highly diverse libraries can be effectively and simply screened for the most growth-advantageous expression solution.

The MoClo vectors constructed in this work were designed to be compatible with the existing (CIDAR) MoClo standard to enable rapid cloning and use in a broad set of applications (see Figure 1). The MoClo destination vectors were constructed using p15A- or pBR322 origins of replication, and either ampicillin (level 0/2), kanamycin (level 1), or chloramphenicol (level 1) resistance markers. The overhang sequences were coded as follows: A (GGAG), E (GCTT), F (CGCT), G (TGCC), and H (ACTA). Level 1 destination vectors were generated with CIDAR MoClo compatible overhangs (A–E, A–F, E–F, F–G, G–H). Inclusion of an “A–F” vector renders these level 1 destination vectors compatible with the broader MoClo standard.^24^ Level 0/2 plasmids carried A–F, E–F, A–G, and A–H overhangs. The nomenclature for these plasmids followed the “DV[A/K/C]_p[15A/BR322/UC19]_[OVERHANG]” standard, with A/K/C indicating antibiotic resistance marker ampicillin/kanamycin/chloramphenicol (respectively), followed by the origin of replication, and finally the two overhang letter codes (e.g., “AE”) (see Table S1). In total, the kit comprises 28 plasmids of which 20 were designated level 1 destination vectors. These vectors feature either a p15A or pBR322 ori and carry either kanamycin or chloramphenicol resistance markers, with various overhang combinations. The remaining 8 plasmids were designed as level 0/2 destination vectors, each carrying either a p15A or pBR322 ori along with an ampicillin resistance cassette, and multiple overhang options (see Table S1 for a comprehensive plasmid list).

To characterize the effect of each origin of replication on expression potential and burden, we constructed a set of 27 GFP constructs (pL1_JRN151_AE to pL1_JRN177_AE), with varying promoters, RBSs and either p15A-, pBR322-, or pUC19-derived origins of replication and a kanamycin resistance marker (DVK_p15A_AE, DVK_pBR322_AE, and DVK_pUC19_AE, respectively). Characterized promoters and RBSs were selected from the CIDAR MoClo kit, consisting of constitutive promoters J23100 (rel. act. 1.0), J23107 (0.47), J23116 (0.16), and RBSs B0034m (rel. act. 1.0), B0032m (0.33) and B0033m (0.01).^17^ We used*E. coli* BW25113 as the host strain for this work, which is derived from*E. coli* K-12 and was the parental strain used for construction of the Keio collection of single gene knockouts.^25^ Since BW25113 is a frequently used host for metabolic engineering studies we aimed to characterize it in terms protein expression capacity and growth burden.

**Figure 2:**
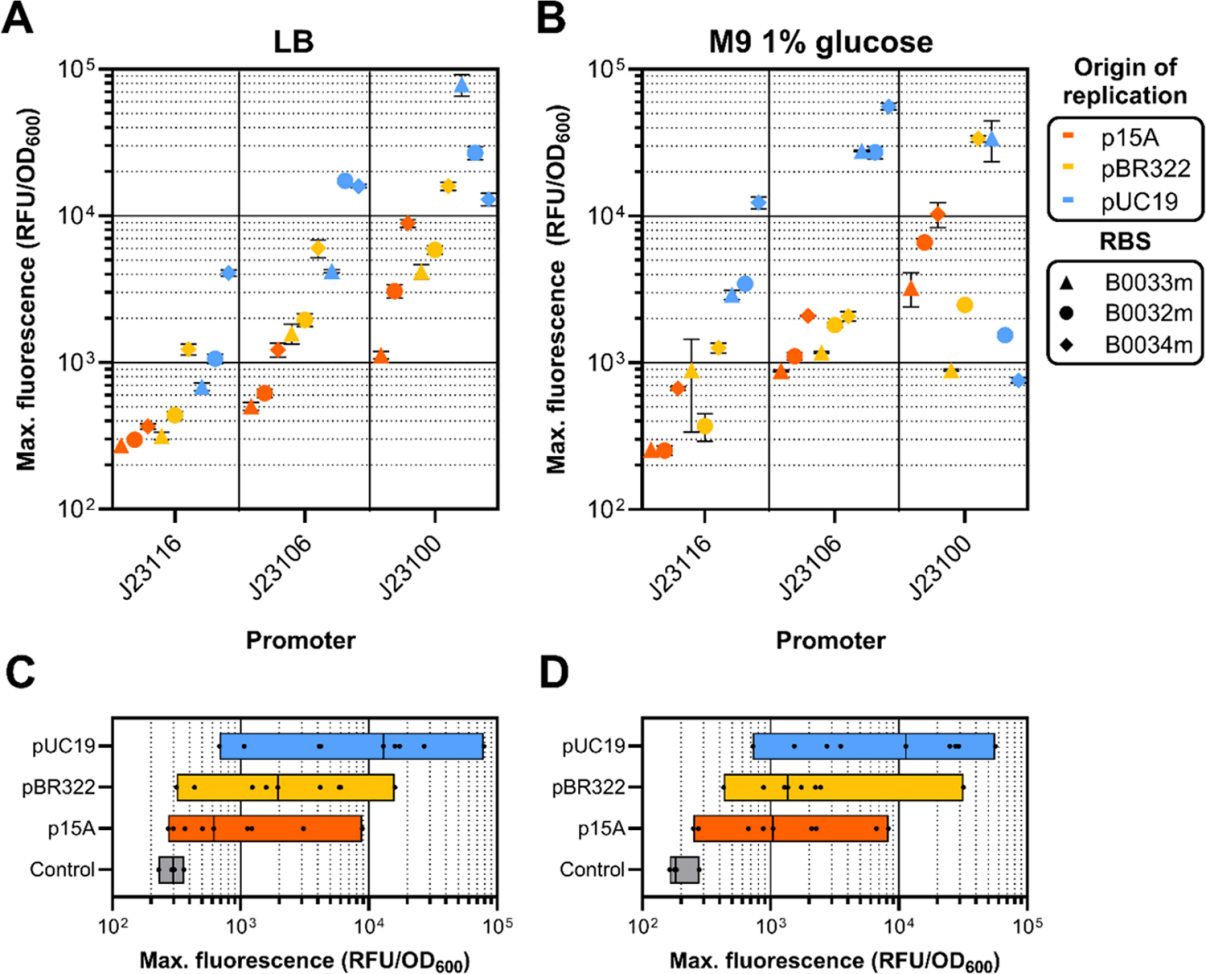
Fluorescence readings of MoClo-constructed GFP constructs. (A) Normalized maximum fluorescence of*E. coli* BW25113 cultures carrying various GFP expression constructs grown in LB or (B) M9 minimal medium with 1% glucose per promoter, RBS, and origin of replication. Error bars represent the standard deviation of three biological replicates. (C) Range of normalized maximum fluorescence measurements of*E. coli* BW25113 cultures grown in LB or (D) M9 minimal medium 1% glucose per origin of replication. Black dots within bars represent individual constructs, black vertical line represents the median normalized maximum fluorescence of all constructs per origin of replication.

**Figure 3:**
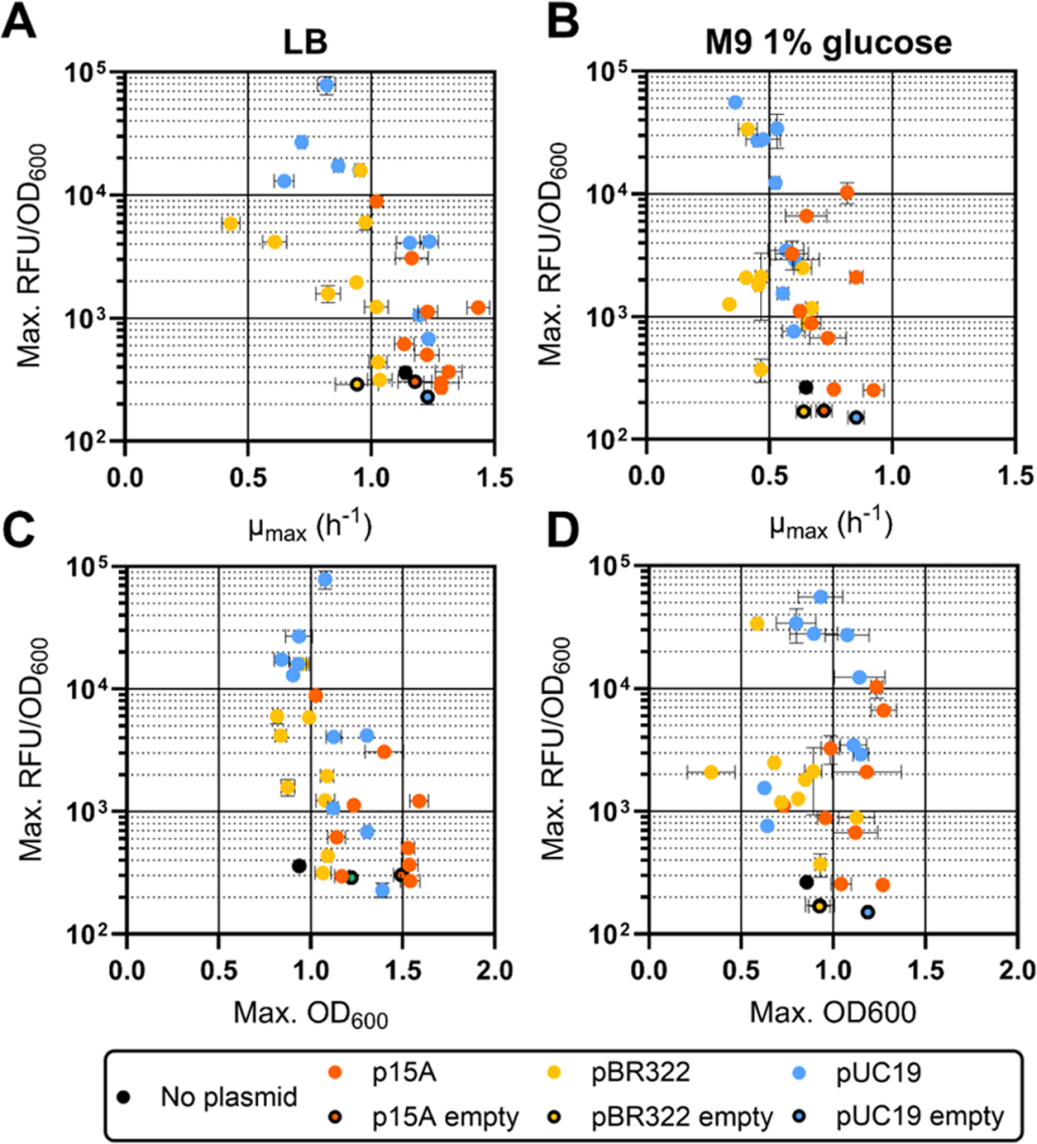
Burden assessment of MoClo-compatible destination vectors with different origins of replication. *E. coli* BW25113 carrying various constitutive GFP expression constructs in p15A, pBR322, or pUC19 backbones were grown in LB (A, C) or M9 minimal medium with 1% glucose (B, D) and assessed for their growth rates (A, B) and maximum achieved OD_600_ (C, D). Data points represent averages of biological triplicate measurements, and error bars represent their standard deviations. The visualized data is also provided in table format in Supporting Table S4, including a distinction of the exact promoter, RBS, and backbone combination used per result.

In general, LB medium resulted in a more consistent relationship between the expected expression strength and measured GFP expression, with stronger promoters, RBSs, and higher copy vectors resulting in a higher GFP output (Figure 2A). Deviation from this pattern occurred only with extremely strong expression constructs using pUC19 backbones in LB medium, which we attributed to growth impairments due to the expression burden, as discussed later (Figure 3A,C). In terms of maximum normalized fluorescence, constructs assembled with a DVK_pUC19_AE backbone consistently achieved the highest values for every tested construct when grown on LB (except for the burdensome J23100/B0034m construct), indicating high expression of GFP even with a low strength promoter and RBS (Figure 2A). At the lower end of the GFP fluorescence spectrum, constructs with p15A-derived origins of replication were dominant. It was not possible to distinguish between the DVK_p15A_AE plasmid encoding a J23116/B0033m GFP construct and an empty vector control, indicating a negligible expression of GFP.

In glucose minimal medium, strains carrying DVK_pBR322_AE as a plasmid backbone displayed somewhat erratic GFP fluorescence profiles, such as J23100/B0033m displaying lower normalized fluorescence compared to J23106/B0033m (Figure 2B). This behavior was mostly caused by the relatively poor growth of the strain when expressing certain constructs (Figure 3B,D). In general, vectors with a pBR322 backbone seemed to confer lower growth rates and lower final OD_600_ values in both LB and glucose minimal medium compared to lower copy p15A and higher copy pUC19 vectors (Figure 3). It is possible that the expression of *rop* and *bom* proteins from the pBR322 origin of replication resulted in unwanted burden in the genetic context of certain GFP expression constructs.

GFP expression from the respective backbones followed an expected trend where higher copy plasmids resulted in higher expression strength, although this profile was more distinctly present when using LB (Figure 2C) compared to M9 glucose medium (Figure 2D). The p15A- and pBR322 backbone plasmids allowed a fine-grained exploration of the expression space, as can be seen from the improved coverage over normalized fluorescence values compared to just using DVK_pUC19_AE as a backbone (Figure 2A,B). Besides improving coverage when using all three backbones, the use of p15A- or pBR322 backbones alone resulted in a smaller range of normalized fluorescence when weak- or medium strength constructs were used compared to the pUC19 backbone (Figure 2A,B, J23116 and J23106). The use of lower-copy plasmids may therefore be beneficial if fine-tuning of constitutive protein expression is required within a specific expression range.

Across all three origins of replications used in this work, the p15A-derived origin of replication demonstrated the most consistent and predictable expression and burden profiles. Constructs using pBR322- and pUC19 backbones displayed irregular growth patterns in minimal medium, especially when high strength promoters and RBSs were used (see Table S4 for μ_max_ and maximum OD_600_ values per construct and condition). This variability resulted in several outliers that deviated in terms of growth rates or final biomass yields (Figure 3B,D). In contrast, strains carrying DVK_p15A_AE backbones exhibited a generally consistent relationship between expression strength and the theoretical strength of each constituent part, together with a relatively low burden profile (Figures 2A,B and Figure 3). Notably, growth rates and final biomass yields of p15A-carrying*E. coli* were consistently equal to or higher than the other two backbones (Figure 3). The maximum normalized fluorescence achieved by GFP constructs in a p15A construct was around 1 × 10^4^ RFU/OD_600_ in both LB and M9 glucose. When compared to GFP expressed from a pUC19 backbone, the maximum RFU/OD_600_ was only ∼8 fold lower in LB and ∼5 fold lower in M9 glucose (Figure 2A,B). Considering the apparent low growth burden that p15A imposes on its host, p15A backbones may therefore be the most suitable choice if reliable profiling of various expression constructs is required.

### Growth-Coupled Optimization of *adhE* Expression by Randomized GGA

Modular cloning not only facilitates the generation of defined constructs with high efficiency, but also allows for efficient randomization of parts while maintaining high fidelity in terms of the order of assembled DNA parts. By including a library of one or more parts in an assembly, the resulting constructs are expected to contain a random distribution of these parts. This approach enables building level 1 constructs with randomized promoters, RBSs, CDSs or terminators. These randomized level 1 constructs can subsequently be used in level 2 assemblies to generate randomized expression constructs of metabolic pathways or enzyme complexes.

If the output of these randomized constructs generates a detectable phenotype, this method can be used to screen for optimal metabolic pathway enzyme expression with relative ease. For example, the heterologous biosynthesis of carotenoids in*E. coli* has been used as a proof-of-principle for this approach, since the resulting color formation in single colonies can be linked to differentially expressed pathway enzymes.^7^ An alternative to this screening approach is to use selection, where the activity of the enzyme(s) of interest contribute to cell fitness, making them growth-coupled. Cell populations carrying a library of growth-coupled enzyme constructs can be used to determine optimal expression levels of the enzyme(s) with relatively ease, either by passaging in selective medium or growing populations in selective continuous cultures. To fully explore the expression space of the enzyme, destination vectors with varying copy numbers should be used to ensure selection for an optimal balance between expression level and plasmid burden.

**Figure 4:**
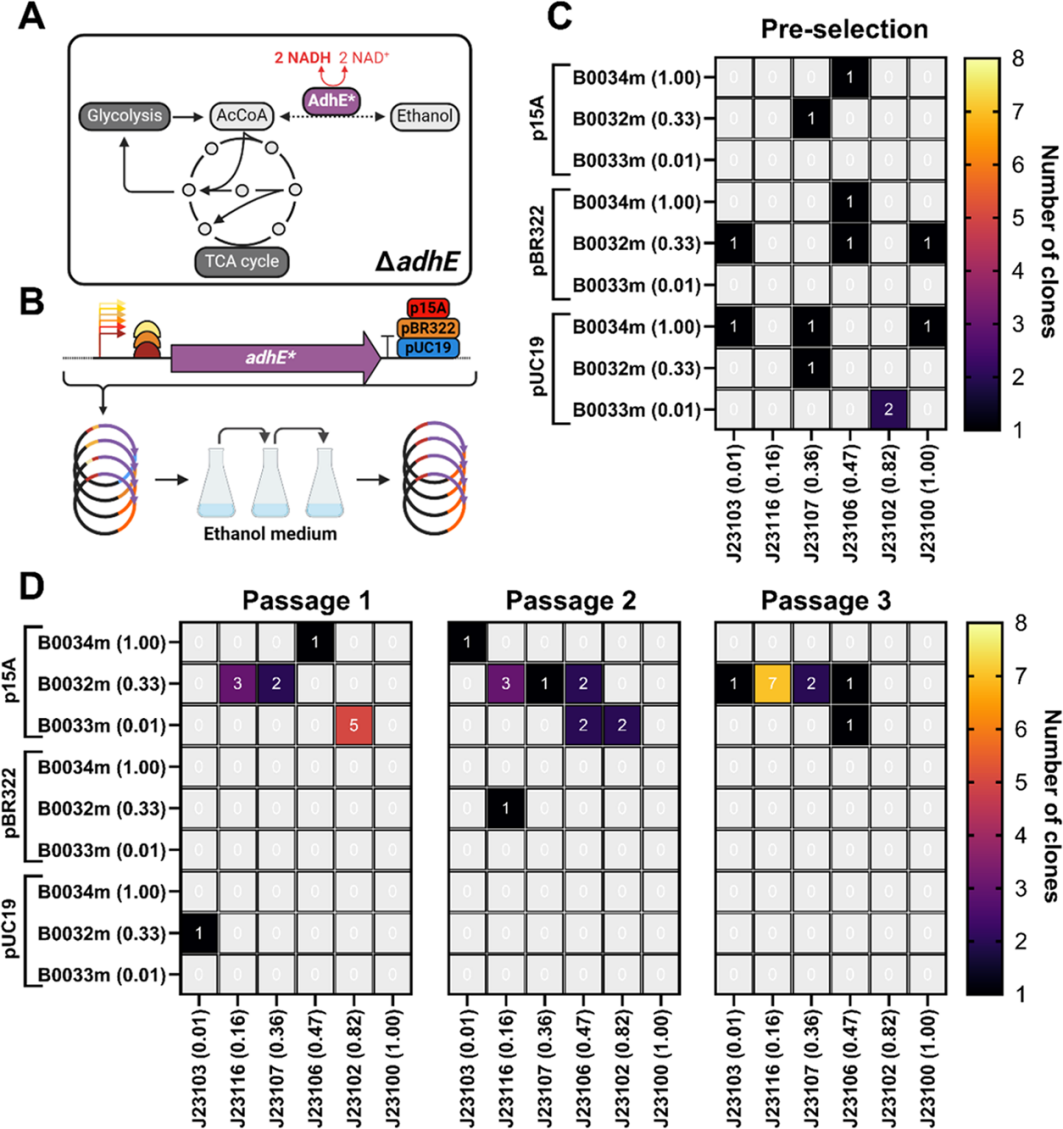
Growth-coupled selection for optimal adhE* expression levels using MoClo-mediated randomized construct assembly. (A) Schematic of*E. coli* central metabolism depicting the catabolism of ethanol by AdhE*—the acetaldehyde intermediate is not depicted. (B) Workflow of the selection experiment starting with randomized Golden Gate Assembly of promoters, RBSs and destination vectors, yielding a diverse population of plasmids. Subsequent transformation and passaging on M9 minimal medium with 1 mM glucose + 300 mM ethanol results in selection for optimal expression of adhE*. (C) Number of sequenced clones carrying a certain adhE* expression construct before selection on ethanol medium was imposed. (D) Number of sequenced clones carrying a certain adhE* expression construct after 1, 2, or 3 passages on M9 minimal medium with 1 mM glucose + 300 mM ethanol. Three independent populations were transformed and passaged, four colonies of each population were picked at each step (pre-selection and passage 1–3) and the resulting construct composition counts were combined in the heatmaps shown in panels C and D. See Table S5 for raw counts and expression construct composition per independent population and per step. Created with Biorender.com.

We demonstrated this concept by optimizing the expression of adhE*, an oxygen-tolerant variant (A267T/E568K) of*E. coli* bifunctional alcohol dehydrogenase *adhE.*(26,27) This enzyme confers*E. coli* the ability to grow on ethanol as a sole carbon source in aerobic environments. To optimize adhE* expression, we constructed a library of various promoters (J23103, J23116, J23107, J23106, J23102, J23100), RBSs (B0033m, B0032m, B0034m) and destination vectors (DVK_pUC19_AE, DVK_pBR322_AE, DVK_p15A_AE), along with the adhE* CDS and a terminator (Figure 4A,B).

The transformed adhE* expression library displayed a random distribution of promoters, RBSs and destination vectors before selection (Figure 4C). Once selection was imposed by supplying ethanol as sole carbon source in M9 minimal medium, a single passage revealed that the majority of isolated clones carried adhE* constructs in a DVK_p15A_AE backbone. This suggested that a plasmid with a low copy origin of replication conferred a growth benefit for the catabolism of ethanol by adhE*. After the third passage, all examined clones carried DVK_p15A_AE backbones and the majority (∼60%) of these expressed adhE* with a J23116 promoter (rel. act. 0.16) and a B0032m RBS (rel. act. 0.33) (Figure 4D). This suggests that relatively low expression levels of adhE* were sufficient to establish optimal growth with ethanol as a carbon source. We observed that each passage (cultures were passaged at OD_600_ ≥ 1) took approximately 48 h to complete (data not shown), which represented a considerably lower growth rate than on glucose. The selection for low adhE* expression levels implicates that the catalytic properties of AdhE* itself were not the limiting factor for growth. In fact, from our initial characterization of the GFP expression constructs in LB and M9 medium (Figure 2A,B) the J23116/B0032m combination in a DVK_p15A_AE backbone led to one of the lowest maximum normalized fluorescence among all tested constructs. This highlighted the importance of providing vectors with a variety of copy numbers in growth-coupled selection experiments to identify truly optimal expression levels. We concluded that the catabolism of ethanol in central metabolism could be limited by its inability to accommodate a higher flux from AdhE*, potentially due to the high levels of NADH produced as a result of ethanol oxidation.

## Discussion

The expression of GFP using various backbones and promoter/RBS combination revealed that on the whole, increasing copy number or promoter/RBS strength resulted in increases in normalized GFP expression, as expected. However, we found that deleterious effects on growth disrupted this pattern at the extremes of expression strength, most notably using pUC19- or pBR322 backbones with strong promoter/RBS parts. The pBR322 backbone also displayed some unexpectedly aberrant burden profiles when using seemingly nominal (i.e., not strong) expression constructs in both LB and M9 glucose minimal medium. We currently do not have an explanation for these observations, but it highlighted that determining the expression- and burden profiles of plasmids before using them in further work is important to ensure the reproducibility of studies. It may also be worthwhile to characterize the expression strength and burden of the plasmids carrying chloramphenicol resistance markers, as changing antibiotic resistance markers was reported to result in differences in expression profiles.^17^

Besides profiling expression strength and burden, the plasmid kit was used in a proof-of-principle experiment to optimize the expression of adhE* for ethanol catabolism in*E. coli*. Since ethanol catabolism in this example is strictly growth-coupled, given it is essential for carbon uptake, the expression of adhE* was expected to be optimized for growth maximization. Indeed, we identified a preferred expression solution after only several rounds of passaging in ethanol-containing medium. Growth-coupled enzymes that are not optimally expressed can lead to deleterious effects on growth.^28^ For example, overconsumption or production of central metabolites may reduce biomass flux, and excess cofactor reduction or oxidation can lead to redox state imbalances. Growth-coupled bioproduction can also benefit from optimal enzyme expression.^29^ By performing enzyme expression selection as described in this work, one or several optimal expression solutions can be identified from large populations of variants with minimal resource requirements.

While performing expression selection with a single enzyme may be unnecessary—since defined constructs can be manually designed and evaluated—the situation changes when multiple enzymes are involved (e.g., in a biosynthetic pathway), as the combinatorial space quickly becomes too large to sample manually. This randomization approach therefore lends itself well to the optimization of enzyme pathway expression, an approach explored in several studies.^7,30−32^ While finding expression optima is the main aim of such an approach, this method also identifies the expression solutions that are selected against, informing and streamlining future design of enzyme expression constructs. By using the vector kit designed in this work for example, optimal expression solutions may be limited to just one backbone type, as was the case with adhE* expression, which may aid the future construction and optimization efforts for single- or dual plasmid systems.

## Conclusions

Golden Gate cloning has quickly become a staple method in synthetic biology research due to its high fidelity, efficiency and ease-of-use compared to other methods such as Gibson- or restriction enzyme cloning. The standardization and characterization of genetic parts, and their public availability, are important aspects for unlocking the full potential of this DNA assembly method.

In this study we constructed and characterized several MoClo-compatible vectors to add to the repertoire of modular parts available for GGA. These vectors were made to be compatible specifically with the CIDAR MoClo kit,^17^ although their overhangs are compatible with the MoClo standard in general.^24^ The effect of plasmid copy number was assessed in terms of GFP expression strength and growth burden, providing useful reference material for those using this set of plasmids, or those using similar plasmids. The addition of lower-copy vectors to the original CIDAR MoClo repertoire allows the assembly of genetic constructs varying up to 500-fold in expression level. The addition of a p15A-derived destination vector also allows construction of multiplasmid systems with compatible origins of replication. Finally, we demonstrated how using plasmids with different origins of replication can help identify optimum expression solutions in growth-coupled designs.

We expect this kit to be useful to synthetic biologists working with*E. coli* expression systems. This work adds to the existing set of publicly available MoClo destination vectors, including the original CIDAR MoClo kit and the more recent broad-host MoClo vector kit,^33^ providing researchers with a wide range of options in terms of expression backbones.

## Data Availability

The destination vectors described in this study are publicly available on Addgene (catalog #217582–217609).

## Supporting Information Available

Tables of plasmids used and constructed in this study; DNA sequences of primers used in this study; sequences of synthesized DNA used in this study; growth parameters of*E. coli* BW25113 transformed with various GFP expression constructs; number and composition of *adhE** expression constructs in picked clones after serial passaging in selective ethanol medium per population and per passaging step (PDF)

## Abbreviations

Ori: origin of replication
MoClo: modular cloning
RBS: ribosome binding site
GGA: golden gate assembly
