# Supplementary material for: Construction and Characterization of MoClo-Compatible Vectors for Modular Protein Expression in E. coli
Source: ACS Synth Biol. 2025 Jan 13;14(2):398–406. doi: 10.1021/acssynbio.4c00564 (PMC11852211; doi:10.1021/acssynbio.4c00564)
Supplement: Supplementary file 1 — sb4c00564_si_001.pdf [file sb4c00564_si_001.pdf]

Supplementary Information

Construction and characterization of MoClo-compatible vectors for modular protein expression in *E. coli*

Jochem R. Nielsen[1], Michael J. Lewis[1], Wei E. Huang[1,*]

[1]Department of Engineering Science, University of Oxford, Oxford, OX1 3PJ, United Kingdom

*Corresponding author: wei.huang@eng.ox.ac.uk and jochem.nielsen@worc.ox.ac.uk

Table S1. Plasmids used and constructed in this study.

Name	Contents	Resistance	Origin of replication	Reference
DVA	*lacZα*	amp^R	pMB1 (pUC19)	[1]
DVK	*lacZα*	kan^R	pMB1 (pUC19)	[1]
pKD3	FRT-cm^R-FRT, oriR6K, amp^R	amp^R,cm^R	R6K gamma	[2]
pSIJ8	araC-P_{ara}-α-β-γ rhaRS-P_{rha}-FLP, amp^R	amp^R	pSC101	[3]
pL0_JRN012_CD	Level 0 part containing domesticated *E. coli* MG1655 *adhE*	amp^R	pMB1 (pUC19)	This study
pL0_JRN017_CD	Level 0 part containing *E. coli* *adhE*^{A267T}	amp^R	pMB1 (pUC19)	This study
pL0_JRN018_CD	Level 0 part containing *E. coli* *adhE*$^{A267T/E568K}$	amp^R	pMB1 (pUC19)	This study
DVK_p15A_AE	*lacZα*	kan^R	p15A	This study
DVK_p15A_AF	*lacZα*	kan^R	p15A	This study
DVK_p15A_EF	*lacZα*	kan^R	p15A	This study
DVK_p15A_FG	*lacZα*	kan^R	p15A	This study
DVK_p15A_GH	*lacZα*	kan^R	p15A	This study
DVC_p15A_AE	*lacZα*	cm^R	p15A	This study
DVC_p15A_AF	*lacZα*	cm^R	p15A	This study
DVC_p15A_EF	*lacZα*	cm^R	p15A	This study
DVC_p15A_FG	*lacZα*	cm^R	p15A	This study
DVC_p15A_GH	*lacZα*	cm^R	p15A	This study
DVK_pBR322_AE	*lacZα*	kan^R	pMB1 (pBR322)	This study
DVK_pBR322_AF	*lacZα*	kan^R	pMB1 (pBR322)	This study
DVK_pBR322_EF	*lacZα*	kan^R	pMB1 (pBR322)	This study
DVK_pBR322_FG	*lacZα*	kan^R	pMB1 (pBR322)	This study
DVK_pBR322_GH	*lacZα*	kan^R	pMB1 (pBR322)	This study
DVC_pBR322_AE	*lacZα*	cm^R	pMB1 (pBR322)	This study
DVC_pBR322_AF	*lacZα*	cm^R	pMB1 (pBR322)	This study
DVC_pBR322_EF	*lacZα*	cm^R	pMB1 (pBR322)	This study
DVC_pBR322_FG	*lacZα*	cm^R	pMB1 (pBR322)	This study
DVC_pBR322_GH	*lacZα*	cm^R	pMB1 (pBR322)	This study
DVA_p15A_AF	*lacZα*	amp^R	p15A	This study
DVA_p15A_AG	*lacZα*	amp^R	p15A	This study

DVA_p15A_EH	*lacZα*	amp^R	p15A	This study
DVA_p15A_AH	*lacZα*	amp^R	p15A	This study
DVA_pBR322_AF	*lacZα*	amp^R	pMB1 (pBR322)	This study
DVA_pBR322_AG	*lacZα*	amp^R	pMB1 (pBR322)	This study
DVA_pBR322_EH	*lacZα*	amp^R	pMB1 (pBR322)	This study
DVA_pBR322_AH	*lacZα*	amp^R	pMB1 (pBR322)	This study
DVK_pUC19_AE	*lacZα*	kan^R	pMB1 (pUC19)	[1]
DVA_pUC19_CD	*lacZα*	amp^R	pMB1 (pUC19)	[1]
DVA_J23100_AB	J23100 promoter part	amp^R	pMB1 (pUC19)	[1]
DVA_J23102_AB	J23102 promoter part	amp^R	pMB1 (pUC19)	[1]
DVA_J23106_AB	J23106 promoter part	amp^R	pMB1 (pUC19)	[1]
DVA_J23107_AB	J23107 promoter part	amp^R	pMB1 (pUC19)	[1]
DVA_J23116_AB	J23116 promoter part	amp^R	pMB1 (pUC19)	[1]
DVA_J23103_AB	J23103 promoter part	amp^R	pMB1 (pUC19)	[1]
DVA_B0034m_BC	B0034m RBS part	amp^R	pMB1 (pUC19)	[1]
DVA_B0032m_BC	B0032m RBS part	amp^R	pMB1 (pUC19)	[1]
DVA_B0033m_BC	B0033m RBS part	amp^R	pMB1 (pUC19)	[1]
DVA_B0015_DE	B0015 terminator	amp^R	pMB1 (pUC19)	[1]
DVA_GFP_CD	Bba_E0040 part, GFP mut3b	amp^R	pMB1 (pUC19)	[1]
pL1_JRN151_AE	J23116_B0033m_GFP_B0015	kan^R	p15A	This study
pL1_JRN152_AE	J23106_B0033m_GFP_B0015	kan^R	p15A	This study
pL1_JRN153_AE	J23100_B0033m_GFP_B0015	kan^R	p15A	This study
pL1_JRN154_AE	J23116_B0032m_GFP_B0015	kan^R	p15A	This study
pL1_JRN155_AE	J23106_B0032m_GFP_B0015	kan^R	p15A	This study
pL1_JRN156_AE	J23100_B0032m_GFP_B0015	kan^R	p15A	This study
pL1_JRN157_AE	J23116_B0034m_GFP_B0015	kan^R	p15A	This study
pL1_JRN158_AE	J23106_B0034m_GFP_B0015	kan^R	p15A	This study
pL1_JRN159_AE	J23100_B0034m_GFP_B0015	kan^R	p15A	This study
pL1_JRN160_AE	J23116_B0033m_GFP_B0015	kan^R	pMB1 (pBR322)	This study
pL1_JRN161_AE	J23106_B0033m_GFP_B0015	kan^R	pMB1 (pBR322)	This study
pL1_JRN162_AE	J23100_B0033m_GFP_B0015	kan^R	pMB1 (pBR322)	This study

pL1_JRN163_AE	J23116_B0032m_GFP_B0015	kan^R	pMB1 (pBR322)	This study
pL1_JRN164_AE	J23106_B0032m_GFP_B0015	kan^R	pMB1 (pBR322)	This study
pL1_JRN165_AE	J23100_B0032m_GFP_B0015	kan^R	pMB1 (pBR322)	This study
pL1_JRN166_AE	J23116_B0034m_GFP_B0015	kan^R	pMB1 (pBR322)	This study
pL1_JRN167_AE	J23106_B0034m_GFP_B0015	kan^R	pMB1 (pBR322)	This study
pL1_JRN168_AE	J23100_B0034m_GFP_B0015	kan^R	pMB1 (pBR322)	This study
pL1_JRN169_AE	J23116_B0033m_GFP_B0015	kan^R	pMB1 (pUC19)	This study
pL1_JRN170_AE	J23106_B0033m_GFP_B0015	kan^R	pMB1 (pUC19)	This study
pL1_JRN171_AE	J23100_B0033m_GFP_B0015	kan^R	pMB1 (pUC19)	This study
pL1_JRN172_AE	J23116_B0032m_GFP_B0015	kan^R	pMB1 (pUC19)	This study
pL1_JRN173_AE	J23106_B0032m_GFP_B0015	kan^R	pMB1 (pUC19)	This study
pL1_JRN174_AE	J23100_B0032m_GFP_B0015	kan^R	pMB1 (pUC19)	This study
pL1_JRN175_AE	J23116_B0034m_GFP_B0015	kan^R	pMB1 (pUC19)	This study
pL1_JRN176_AE	J23106_B0034m_GFP_B0015	kan^R	pMB1 (pUC19)	This study
pL1_JRN177_AE	J23100_B0034m_GFP_B0015	kan^R	pMB1 (pUC19)	This study

Table S2. DNA sequences of primers used in this study.

**Primer name**	**Sequence**	**Purpose**
JRN189	CTGGCGCTGCGCTTT	Introduce E568K into adhE
JRN190	TTCGAAGTGAGTTTCCGGATG	Introduce E568K into adhE
JRN191	ATCCGGAAACTCACTTCGAAAAGCTGGCGCTGCGCTTTATGGA	adhE E568K oligo bridge
JRN224	CGGCTATCTGTTGCAGG	Introduce A267T into adhE
JRN225	AACGTTCACGTACAGCGTC	Introduce A267T into adhE
JRN226	GTTGACTCTGTTTATGACGCTGTACGTGAACGTTTTACCACCCA CGGCGGCTATCTGTTGCAGGGTAAAGAGC	adhE A267T oligo bridge
JRN273	gctaaggatgatttctgg	Check MoClo vector insert
JRN274	agtgagcgaggaagc	Check MoClo vector insert
JRN279	AGTCGAAGACGAAATGGCTGTTACTAATGTCGCTG	Domesticate *adhE*
JRN280	AGTCGAAGACCAGTCATCTTCAGACAGAACACCAC	Domesticate *adhE*
JRN281	AGTCGAAGACGTTGACACTTTTGGTACCATCA	Domesticate *adhE*
JRN282	AGTCGAAGACGTGTTTTCAGACTGATCAGCGA	Domesticate *adhE*
JRN283	AGTCGAAGACCAAAACCCGTAACGCCATTATCT	Domesticate *adhE*
JRN284	AGTCGAAGACCATTCGAAATCTTTAGCGCG	Domesticate *adhE*
JRN285	AGTCGAAGACAGCGAAGATGCGGTAGAAAAAGCAGA	Domesticate *adhE*
JRN286	AGTCGAAGACAGAAGAACACTTCAGTTTCAACGC	Domesticate *adhE*
JRN287	AGTCGAAGACGATCTTCGAAGTAGAAGCGG	Domesticate *adhE*

JRN288	AGTCGAAGACGAACCTTTAAGCGGATTTTTTCGCT	Domesticate *adhE*
JRN386	Ctgttgtggaagccgttatag	Check *adhE* locus
JRN387	Ctaatgtttaaactcttttagtaaatcacag	Check *adhE* locus
JRN384	CCGTTTATGTTGCCAGACAGCGCTACTGATTAAGCGGATTTTTTCGCTTTtgtgtaggctggagctgcttc	Amplify cmR marker with adhE flanks from pKD3
JRN385	CGAGCAGATGATTTACTAAAAAAGTTTAACATTATCAGGAGAGCATTATGcatatgaatatcctccttag	Amplify cmR marker with adhE flanks from pKD3
JRN413	GTCGTCTCCtggaagacatGGAGagagacctgGGAGACGAC	Oligo_A_L1_TV_fwd
JRN414	GTCGTCTCCgggtctctGCTTatgtcttctGGAGACGAC	Oligo_E_L1_SU_fwd
JRN415	GTCGTCTCCtggaagacatGCTTagagacctgGGAGACGAC	Oligo_E_L1_TV_fwd
JRN416	GTCGTCTCCgggtctctCGCTatgtcttctGGAGACGAC	Oligo_F_L1_SU_fwd
JRN417	GTCGTCTCCtggaagacatCGCTagagacctgGGAGACGAC	Oligo_F_L1_TV_fwd
JRN418	GTCGTCTCCgggtctctTGCCatgtcttctGGAGACGAC	Oligo_G_L1_SU_fwd
JRN419	GTCGTCTCCtggaagacatTGCCagagacctgGGAGACGAC	Oligo_G_L1_TV_fwd
JRN420	GTCGTCTCCgggtctctACTAatgtcttctGGAGACGAC	Oligo_H_L1_SU_fwd
JRN421	GTCGTCTCCtggaggtctcaGGAGatgtcttccctgGGAGACGAC	Oligo_A_L2_TV_fwd
JRN422	GTCGTCTCCtggaggtctcaGCTTatgtcttccctgGGAGACGAC	Oligo_E_L2_TV_fwd
JRN423	GTCGTCTCCgggttaagaagacgtCGCTagagaccttctGGAGACGAC	Oligo_F_L2_SU_fwd
JRN424	GTCGTCTCCtggaggtctcaCGCTatgtcttccctgGGAGACGAC	Oligo_F_L2_TV_fwd
JRN425	GTCGTCTCCgggttaagaagacgtTGCCagagaccttctGGAGACGAC	Oligo_G_L2_SU_fwd
JRN426	GTCGTCTCCgggttaagaagacgtACTAagagaccttctGGAGACGAC	Oligo_H_L2_SU_fwd
JRN427	GTCGTCTCCcaggtctctCTCCatgtcttccaGGAGACGAC	Oligo_A_L1_TV_rev
JRN428	GTCGTCTCCagaagacatAAGCagagacccGGAGACGAC	Oligo_E_L1_SU_rev
JRN429	GTCGTCTCCcaggtctctAAGCatgtcttccaGGAGACGAC	Oligo_E_L1_TV_rev
JRN430	GTCGTCTCCagaagacatAGCGagagacccGGAGACGAC	Oligo_F_L1_SU_rev
JRN431	GTCGTCTCCcaggtctctAGCGatgtcttccaGGAGACGAC	Oligo_F_L1_TV_rev
JRN432	GTCGTCTCCagaagacatGGCAagagacccGGAGACGAC	Oligo_G_L1_SU_rev
JRN433	GTCGTCTCCcaggtctctGGCAatgtcttccaGGAGACGAC	Oligo_G_L1_TV_rev
JRN434	GTCGTCTCCagaagacatTAGTagagacccGGAGACGAC	Oligo_H_L1_SU_rev
JRN435	GTCGTCTCCcagggaagacatCTCCtgagacctccaGGAGACGAC	Oligo_A_L2_TV_rev
JRN436	GTCGTCTCCcagggaagacatAAGCtgagacctccaGGAGACGAC	Oligo_E_L2_TV_rev
JRN437	GTCGTCTCCagaaggtctctAGCGacgtcttcttaacccGGAGACGAC	Oligo_F_L2_SU_rev
JRN438	GTCGTCTCCcagggaagacatAGCGtgagacctccaGGAGACGAC	Oligo_F_L2_TV_rev
JRN439	GTCGTCTCCagaaggtctctGGCAacgtcttcttaacccGGAGACGAC	Oligo_G_L2_SU_rev
JRN440	GTCGTCTCCagaaggtctctTAGTacgtcttcttaacccGGAGACGAC	Oligo_H_L2_SU_rev

Table S3. Sequences of synthesized DNA used in this study.

Name	Sequence
p15A origin of replication fragment	TTCGTCTCCTTCTGGATCGGTTGTCGAGTAAGGATCTCCAGGCATCAAATAAAACGAAAGGCTCAGTCGAAAGACTGGGCCTTTCGTTTTATCTGTTGTTTGTCGGTGAACGCTCTCTACTAGAGTCACACTGGCTCACCTTCGGGTGGGCCTTTCTGCGTTTATAGGATCCTAACTCGAGCCTAGGGATATATTCCGCTTCCTCGCTCACTGACTCGCTACGCTCGGTCGTTCGACTGCGGCGAGCGGAAATGGCTTACGAACGGGGCGGAGATTTCCTGGAAGATGCCAGGAAGATACTTAACAGGGAAGTGAGAGGGCCGCGGCAAAGCCGTTTTTCCATAGGCTCCGCCCCCCTGACAAGCATCACGAAATCTGACGCTCAAATCAGTGGTGGCGAAACCCGACAGGACTATAAAGATACCAGGCGTTTCCCCCTGGCGGCTCCCTCGTGCGCTCTCCTGTTCCTGCCTTTCGGTTTACCGGTGTCATTCCGCTGTTATGGCCGCGTTTGTCTCATTCCACGCCTGACACTCAGTTCCGGGTAGGCAGTTCGCTCCAAGCTGGACTGTATGCACGAACCCCCCGTTCAGTCCGACCGCTGCGCCTTATCCGGTAACTATCGTCTTGAGTCCAACCCGGAAAGACATGCAAAAGCACCACTGGCAGCAGCCACTGGTAATTGATTTAGAGGAGTTAGTCTTGAAGTCATGCGCCGGTTAAGGCTAAACTGAAAGGACAAGTTTTGGTGACTGCGCTCCTCCAAGCCAGTTACCTCGGTTCAAAGAGTTGGTAGCTCAGAGAACCTTCGAAAAACCGCCCTGCAAGGCGGTTTTTTCGTTTTCAGAGCAAGAGATTACGCGCAGACCAAAACGATCTCAAGAAGATCATCTTATTAATCAGATAAAATATTTCT

	AGATTTCAGTGCAATTTATCTCTTCAAATGTAGCACCTGAAGTCAGCCCCATACGATATAAGT<br>TGTTACTAGTGCTTGGATTCTCACCAATAAAAAACGCCCGGCGGCAACCGAGCGTTCTGAA<br>CAAATCCAGATGGAGTTCTGAGGTCATTACTGGATCTATCAACAGGAGTCCAAGCGAGCTC<br>TCGAACCCCAGAGTCCCGCGATTGGAGACGAC
pBR322 origin of replication fragment	TTCGTCTCCTTCTAACACCTACATCTGTATTAACGAAGCGCTGGCATTGACCCTGAGTGATTT<br>TTCTCTGGTCCCGCCGCATCCATACCGCCAGTTGTTTACCCTCACAACGTTCCAGTAACCG<br>GGCATGTTCATCATCAGTAACCCGTATCGTGAGCATCCTCTCTCGTTTCATCGGTATCATTAC<br>CCCATGAACAGAAATCCCCCTTACACGGAGGCATCAGTGACCAAACAGGAAAAAACCGCC<br>CTTAACATGGCCCGCTTTATCAGAAGCCAGACATTAACGCTTCTGGAGAAACTCAACGAGCT<br>GGACGCGGATGAACAGGCAGACATCTGTGAATCGCTTCACGACCACGCTGATGAGCTTTAC<br>CGCAGCTGCCTCGCGCGTTTCGGTGATGACGGTGAAAACCTCTGACACATGCAGCTCCCG<br>GCGACGGTCACAGCTTGTCTGTAAGCGGATGCCGGGAGCAGACAAGCCCGTCAGGGCGC<br>GTCAGCGGGTGTTGGCGGGTGTCGGGGCGCAGCCATGACCCAGTCACGTAGCGATAGCG<br>GAGTGTATACTGGCTTAACTATGCGGCATCAGAGCAGATTGTACTGAGAGTGCACCATTGCG<br>GTGTGAAATACCGCACAGATGCGTAAGGAGAAAATACCGCATCAGGCGCTCTTCCGCTTCC<br>TCGCTCACTGACTCGCTGCGCTCGGTCGTTCGGCTGCGGCGAGCGGTATCAGCTCACTCA<br>AAGGCGGTAATACGGTTATCCACAGAATCAGGGGATAACGCAGGAAAGAACATGTGAGCAA<br>AAGGCCAGCAAAAGGCCAGGAACCGTAAAAAGGCCGCGTTGCTGGCGTTTTTCCATAGGC<br>TCCGCCCCCCTGACGAGCATCACAAAAATCGACGCTCAAGTCAGAGGTGGCGAAACCCGA<br>CAGGACTATAAAGATACCAGGCGTTTCCCCCTGGAAGCTCCCTCGTGCGCTCTCCTGTTCC<br>GACCCTGCCGCTTACCGGATACCTGTCCGCCTTTCTCCCTTCGGGAAGCGTGGCGCTTTC<br>TCATAGCTCACGCTGTAGGTATCTCAGTTCGGTGTAGGTCGTTCGCTCCAAGCTGGGCTGT<br>GTGCACGAACCCCCCGTTCAGCCCGACCGCTGCGCCTTATCCGGTAACTATCGTCTTGAGT<br>CCAACCCGGTAAGACACGACTTATCGCCACTGGCAGCAGCCACTGGTAACAGGATTAGCA<br>GAGCGAGGTATGTAGGCGGTGCTACAGAGTTCTTGAAGTGGTGGCCTAACTACGGCTACAC<br>TAGAAGGACAGTATTTGGTATCTGCGCTCTGCTGAAGCCAGTTACCTTCGGAAAAAGAGTT<br>GGTAGCTCTTGATCCGGCAAACAAACCACCGCTGGTAGCGGTGGTTTTTTTGTTTGCAAGC<br>AGCAGATTACGCGCAGAAAAAAAGGATCTCAAGAAGATCCTTTGATCTTTTCTACCGGTCCG<br>TCCAAAAAAAAAGGCTCCAAAAGGAGCCTTTAATTGTATCGGTGATTGGAGACGAC
amp^R fragment	TTCGTCTCCGATTACCAATGCTTAATCAGTGAGGCACCTATCTCAGCGATCTGTCTATTTCGT<br>TCATCCATAGTTGCCTGACTCCCCGTCGTGTAGATAACTACGATACGGGAGGGCTTACCATC<br>TGGCCCCAGTGCTGCAATGATACCGCGCGACCCACGCTCACCGGCTCCAGATTTATCAGC<br>AATAAACCAGCCAGCCGGAAGGGCCGAGCGCAGAAGTGGTCCTGCAACTTTATCCGCCTC<br>CATCCAGTCTATTAATTGTTGCCGGGAAGCTAGAGTAAGTAGTTCGCCAGTTAATAGTTTGC<br>GCAACGTTGTTGCCATTGCTACAGGCATCGTGGTGTCACGCTCGTCGTTTGGTATGGCTTC<br>ATTCAGCTCCGGTTCCCAACGATCAAGGCGAGTTACATGATCCCCCATGTTGTGCAAAAAA<br>GCGGTTAGCTCCTTCGGTCCTCCGATCGTTGTCAGAAGTAAGTTGGCCGCAGTGTTATCAC<br>TCATGGTTATGGCAGCACTGCATAATTCTCTTACTGTCATGCCATCCGTAAGATGCTTTTCTG<br>TGACTGGTGAGTACTCAACCAAGTCATTCTGAGAATAGTGTATGCGGCGACCGAGTTGCTC<br>TTGCCCGGCGTCAATACGGGATAATACCGCGCCACATAGCAGAACTTTAAAAGTGCTCATCA<br>TTGGAAAACGTTCTTCGGGGCGAAAACTCTCAAGGATCTTACCGCTGTTGAGATCCAGTTC<br>GATGTAACCCACTCGTGCACCCAACTGATCTTCAGCATCTTTTACTTTCACCAGCGTTTCTG<br>GGTGAGCAAAAACAGGAAGGCAAAATGCCGCAAAAAAGGGAATAAGGGCGACACGGAAAT<br>GTTGAATACTCATACTCTTCCTTTTTCAATATTATTGAAGCATTTATCAGGGTTATTGTCTCATG<br>AGCGGATACATATTTGAATGTATTTAGAAAAATAAACAAATAGGGGTTCCGCGCACATTTCCC<br>CGAAAAGTGCCACCTGACGTCTAAGAAACCATTATTATCATGACATTAACCTATAAAAATAGG<br>CGTATCACGAGGCAGAATTTCAGATAAAAAAAATCCTTAGCTTTCGCTAAGGATGATTTCTGG<br>AATTCGCGGCCGCTTCTAGAGACTAGTGGAGGAGACGAC
kan^R fragment	TTCGTCTCCGATTAGAAAAACTCATCGAGCATCAAATGAAACTGCAATTTATTCATATCAGGA<br>TTATCAATACCATATTTTTGAAAAAGCCGTTTCTGTAATGAAGGAGAAAACTCACCGAGGCAG<br>TTCCATAGGATGGCAAGATCCTGGTATCGGTCTGCGATTCCGACTCGTCCAACATCAATACA<br>ACCTATTAATTTCCCCTCGTCAAAAATAAGGTTATCAAGTGAGAAATCACCATGAGTGACGAC<br>TGAATCCGGTGAGAATGGCAAAAGCTTATGCATTTCTTTCCAGACTTGTTCAACAGGCCAGC<br>CATTACGCTCGTCATCAAAATCACTCGCATCAACCAAACCGTTATTCATTCGTGATTGCGCCT<br>GAGCAAGACGAAATACGCGATCGCTGTTAAAAGGACAATTACAAACAGGAATCGAATGCAA<br>CCGGCGCAGGAACACTGCCAGCGCATCAACAATATTTTCACCTGAATCAGGATATTCTTCTA<br>ATACCTGGAATGCTGTTTTCCCGGGGATCGCAGTGGTGAGTAACCATGCATCATCAGGAGT<br>ACGGATAAAATGCTTGATGGTCGGAAGAGGCATAAATTCCGTCAGCCAGTTTAGTCTGACCA

	TCTCATCTGTAACATCATTGGCAACGCTACCTTTGCCATGTTTCAGAAACAACTCTGGCGCATCGGGCTTCCCATACAATCGATAGATTGTCGCACCTGATTGCCCGACATTATCGCGAGCCCATTTATACCCATATAAATCAGCATCCATGTTGGAATTTAATCGCGGCCTGGAGCAAGACGTTTCCCGTTGAATATGGCTCATAACACCCCTTGTATTACTGTTTATGTAAGCAGACAGTTTTATTGTTCATGATGATATATTTTTATCTTGTGCAATGTAACATCAGAGATTTTGAGACACAACGTGGCTTTGTTGAATAAATCGAACTTTTGCTGAGTTGAAGGATCAGCTCGAGTGCCACCTGACGTCTAAGAAACCATTATTATCATGACATTAACCTATAAAAATAGGCGTATCACGAGGCAGAATTTCAGATAAAAAAAATCCTTAGCTTTCGCTAAGGATGATTTCTGGAATTCGCGGCCGCTTCTAGAGACTAGTGGAGGAGACGAC
cm^R fragment	TTCGTCTCCGATTACGCCCCGCCCTGCCACTCATCGCAGTACTGTTGTAATTCATTAAGCATTCTGCCGACATGGAAGCCATCACAAACGGCATGATGAACCTGAATCGCCAGCGGCATCAGCACCTTGTCGCCTTGCGTATAATATTTGCCCATGGTGAAAACGGGGGCGAAGAAGTTGTCCATATTGGCCACGTTTAAATCAAAACTGGTGAAACTCACCCAGGGATTGGCTGATACGAAAAACATATTCTCAATAAACCCTTTAGGGAAATAGGCCAGGTTTTCACCGTAACACGCCACATCTTGCGAATATATGTGTAGAAACTGCCGGAAATCGTCGTGGTATTCACTCCAGAGCGATGAAAACGTTTCAGTTTGCTCATGGAAAACGGTGTAACAAGGGTGAACACTATCCCATATCACCAGCTCACCGTCTTTCATTGCCATACGTAATTCCGGATGAGCATTCATCAGGCGGGCAAGAATGTGAATAAAGGCCGGATAAAACTTGTGCTTATTTTTCTTTACGGTCTTTAAAAAGGCCGTAATATCCAGCTGAACGGTCTGGTTATAGGTACATTGAGCAACTGACTGAAATGCCTCAAAATGTTCTTTACGATGCCATTGGGATATATCAACGGTGGTATATCCAGTGATTTTTTTCTCCATTTTAGCTTCCTTAGCTCCTGAAAATCTCGACAACTCAAAAAATACGCCCGGTAGTGATCTTATTTCATTATGGTGAAAGTTGGAACCTCTTACGTGCCGATCATGCCACCTGACGTCTAAGAAACCATTATTATCATGACATTAACCTATAAAAATAGGCGTATCACGAGGCAGAATTTCAGATAAAAAAAATCCTTAGCTTTCGCTAAGGATGATTTCTGGAATTCGCGGCCGCTTCTAGAGACTAGTGTGGAGGAGACGAC
lacZα fragment	GTCGTCTCCCCTGCACCATATGCGGTGTGAAATACCGCACAGATGCGTAAGGAGAAAATACCGCATCAGGCGCCATTCGCCATTCAGGCTGCGCAACTGTTGGGAAGGGCGATCGGTGCGGGCCTCTTCGCTATTACGCCAGCTGGCGAAAGGGGGATGTGCTGCAAGGCGATTAAGTTGGGTAACGCCAGGGTTTTCCCAGTCACGACGTTGTAAAACGACGGCCAGTGAATTCGAGCTCGGTACCCGGGGATCCTCTAGAGTCGACCTGCAGGCATGCAAGCTTGGCGTAATCATGGTCATAGCTGTTTCCTGTGTGAAATTGTTATCCGCTCACAATTCCACACAACATACGAGCCGGAAGCATAAAGTGTAAAGCCTGGGGTGCCTAATGAGTGAGCTAACTCACATTAATTGCGTTGCGCTCACTGCCCGCTTTCCAGTCGGGAAACCTGTCGTGCCAGCTGCATTAATGAATCGGCCAACGCGCGGGGGTGGAGACGAC

Table S4. Growth parameters of *E. coli* BW25113 transformed with various GFP expression constructs. Strains were cultivated either in M9 minimal medium with 1% glucose, or rich LB medium. Values for maximum growth rates (μ_{max}) and maximum OD_{600} represent averages of biological triplicate measurements, and their respective standard deviations are provided.

			M9 1% glucose medium				LB medium			
**Backbone**	**RBS**	**Promoter**	μ_{max} (h^{-1})	Stdev (h^{-1})	Max. OD_{600}	Stdev	μ_{max} (h^{-1})	Stdev (h^{-1})	Max. OD_{600}	Stdev
No plasmid			0.649	0.0246	0.857	0.0153	1.14	0.0138	0.938	0.00821
p15A empty			0.723	0.0322	0.927	0.0781	1.18	0.0687	1.49	0.0421
pBR322 empty			0.639	0.0300	0.925	0.0583	0.942	0.0876	1.22	0.0159
pUC19 empty			0.853	0.0325	1.19	0.0171	1.23	0.0155	1.39	0.0253
**p15A**	**B0033m**	**J23116**	0.763	0.0101	1.04	0.0562	1.28	0.0151	1.54	0.0514
		**J23106**	0.671	0.0389	0.958	0.0435	1.23	0.0494	1.53	0.0356
		**J23100**	0.592	0.0676	0.985	0.0499	1.23	0.0392	1.23	0.00458
	**B0032m**	**J23116**	0.924	0.0415	1.27	0.0105	1.28	0.0709	1.17	0.0412
		**J23106**	0.623	0.0139	0.731	0.0208	1.13	0.0397	1.14	0.0478
		**J23100**	0.651	0.0838	1.27	0.0685	1.16	0.0661	1.40	0.104
	**B0034m**	**J23116**	0.738	0.0741	1.12	0.119	1.31	0.0535	1.54	0.0457
		**J23106**	0.854	0.0259	1.18	0.188	1.43	0.0458	1.59	0.0499
		**J23100**	0.815	0.0126	1.24	0.0314	1.02	0.0264	1.03	0.0281
**pBR322**	**B0033m**	**J23116**	0.466	0.0232	0.891	0.0466	1.03	0.0497	1.07	0.0432
		**J23106**	0.672	0.0244	0.718	0.0200	0.824	0.0493	0.874	0.0369
		**J23100**	0.655	0.0214	1.13	0.0956	0.607	0.0484	0.836	0.0298
	**B0032m**	**J23116**	0.464	0.0119	0.930	0.0266	1.03	0.0357	1.09	0.0204
		**J23106**	0.455	0.0151	0.848	0.0147	0.940	0.00255	1.09	0.0357
		**J23100**	0.636	0.0355	0.680	0.0172	0.429	0.0364	0.991	0.0172
	**B0034m**	**J23116**	0.337	0.0079	0.808	0.0114	1.02	0.0482	1.08	0.0512
		**J23106**	0.405	0.00773	0.338	0.130	0.976	0.0166	0.818	0.0222
		**J23100**	0.411	0.0382	0.587	0.0184	0.956	0.00913	0.951	0.0405
**pUC19**	**B0033m**	**J23116**	0.603	0.100	1.15	0.0414	1.23	0.00895	1.31	0.0189
		**J23106**	0.474	0.0691	0.894	0.1282	1.24	0.0372	1.30	0.0153
		**J23100**	0.531	0.0131	0.799	0.107	0.818	0.0349	1.08	0.0104
	**B0032m**	**J23116**	0.567	0.0723	1.11	0.0720	1.20	0.0237	1.12	0.0277
		**J23106**	0.451	0.0169	1.08	0.119	0.865	0.0190	0.841	0.0401
		**J23100**	0.554	0.0236	0.626	0.00424	0.717	0.0151	0.936	0.0715
	**B0034m**	**J23116**	0.524	0.0167	1.14	0.138	1.16	0.0547	1.12	0.0423
		**J23106**	0.361	0.00275	0.931	0.1203	0.951	0.0190	0.931	0.0446
		**J23100**	0.599	0.0462	0.642	0.00190	0.647	0.0407	0.904	0.00473

Table S5. Number of colonies carrying a certain *adhE expression construct after serial passaging in selective ethanol medium.** Three independent populations (Pop. 1-3) were serially passaged and the plasmid insert of four clones per population sequenced before passaging and after each passage. The data represents count data of each specific construct carrying either a p15A, pBR322 or pUC19 origin of replication, a promoter variant (J23103/J23116/J23107/J23106/J23102/J23100) and an RBS variant (B0033m, B0032m, B0034m).

	**Pre-passage**			**Passage 1**			**Passage 2**			**Passage 3**		
	**Pop. 1**	**Pop. 2**	**Pop. 3**	**Pop. 1**	**Pop. 2**	**Pop. 3**	**Pop. 1**	**Pop. 2**	**Pop. 3**	**Pop. 1**	**Pop. 2**	**Pop. 3**
**p15A**	**1** (J23106-B0034m)	**1** (J23107-B0032m)		**1** (J23107-B0032m)<br>**2** (J23102-B0033m)	**2** (J23116-B0032m)<br>**1** (J23107-B0032m)<br>**1** (J23102-B0033m)	**1** (J23116-B0032m)<br>**1** (J23106-B0034m)<br>**2** (J23102-B0033m)	**1** (J23116-B0032m)<br>**1** (J23106-B0033m)<br>**2** (J23102-B0033m)	**1** (J23103-B0034m)<br>**1** (J23116-B0032m)<br>**1** (J23107-B0032m)<br>**1** J23106-B0032m)	**1** (J23116-B0032m)<br>**1** (J23106-B0032m)<br>**1** (J23106-B0033m)	**3** (J23116-B0032m)<br>**1** (J23106-B0032m)	**2** (J23116-B0032m)<br>**1** (J23107-B0032m)<br>**1** (J23106-B0033m)	**1** (J23103-B0032m)<br>**2** (J23116-B0032m)<br>**1** (J23107-B0032m)
**pBR322**	**1** (J23106-B0032m)	**1** (J23100-B0032m)	**1** (J23103-B0032m)<br>**1** (J23106-B0034m)						**1** (J23116-B0032m)			
**pUC19**	**1** (J23107-B0034m)<br>**1** (J23100-B0034m)	**1** (J23102-B0033m)<br>**1** (J23103-B0034m)	**1** (J23102-B0033m)<br>**1** (J23107-B0032m)	**1** (J23103-B0032m)								

References

1. Iverson, S. V., Haddock, T. L., Beal, J., and Densmore, D. M. (2016) CIDAR MoClo: Improved MoClo Assembly Standard and New E. coli Part Library Enable Rapid Combinatorial Design for Synthetic and Traditional Biology. *ACS Synth Biol 5*, 99–103.

2. Datsenko, K. A., and Wanner, B. L. (2000) One-step inactivation of chromosomal genes in Escherichia coli K-12 using PCR products. *Proceedings of the National Academy of Sciences 97*, 6640–6645.

3. Jensen, S. I., Lennen, R. M., Herrgård, M. J., and Nielsen, A. T. (2015) Seven gene deletions in seven days: Fast generation of Escherichia coli strains tolerant to acetate and osmotic stress. *Sci Rep 5*, 17874.
